# Statistical analysis plan for evaluating low‐ vs. standard‐dose alteplase in the ENhanced Control of Hypertension and Thrombolysis strokE stuDy (ENCHANTED)

**DOI:** 10.1111/ijs.12602

**Published:** 2015-08-18

**Authors:** Craig S. Anderson, Mark Woodward, Hisatomi Arima, Xiaoying Chen, Richard I. Lindley, Xia Wang, John Chalmers, ENCHANTED Investigators

**Affiliations:** ^1^ The George Institute for Global HealthRoyal Prince Alfred Hospital and University of SydneySydneyNSWAustralia; ^2^ Centre for Epidemiologic Research in AsiaShiga University of Medical SciencesShiga UniversityShigaJapan

**Keywords:** acute, alteplase, clinical trials, statistical analysis plan, stroke, thrombolysis

## Abstract

**Background:**

The ENhanced Control of Hypertension And Thrombolysis strokE stuDy trial is a 2 × 2 quasi‐factorial active‐comparison, prospective, randomized, open, blinded endpoint clinical trial that is evaluating in thrombolysis‐eligible acute ischemic stroke patients whether: (1) low‐dose (0·6 mg/kg body weight) intravenous alteplase has noninferior efficacy and lower risk of symptomatic intracerebral hemorrhage compared with standard‐dose (0·9 mg/kg body weight) intravenous alteplase; and (2) early intensive blood pressure lowering (systolic target 130–140 mmHg) has superior efficacy and lower risk of any intracerebral hemorrhage compared with guideline‐recommended blood pressure control (systolic target <180 mmHg).

**Objective:**

To outline in detail the predetermined statistical analysis plan for the ‘alteplase dose arm’ of the study.

**Methods:**

All data collected by participating researchers will be reviewed and formally assessed. Information pertaining to the baseline characteristics of patients, their process of care, and the delivery of treatments will be classified, and for each item, appropriate descriptive statistical analyses are planned with appropriate comparisons made between randomized groups. For the trial outcomes, the most appropriate statistical comparisons to be made between groups are planned and described.

**Results:**

A statistical analysis plan was developed for the results of the alteplase dose arm of the study that is transparent, available to the public, verifiable, and predetermined before completion of data collection.

**Conclusions:**

We have developed a predetermined statistical analysis plan for the ENhanced Control of Hypertension And Thrombolysis strokE stuDy alteplase dose arm which is to be followed to avoid analysis bias arising from prior knowledge of the study findings.

Alteplase is the recombinant form of the naturally occurring tissue plasminogen activator (t‐PA) that is produced by endothelial and other vascular cells. It was the first recombinant tissue plasminogen activator (rt‐PA) to be licensed for the management of various thromboembolic diseases including acute myocardial infarction in 1987, massive pulmonary embolism in 1990, and acute ischemic stroke (AIS) in 1996. The basis of the latter license was the pivotal National Institutes of Neurological Diseases and Stroke (NINDS) trial 1, where use of intravenous (iv) alteplase at a dose of 0·9 mg/kg body weight (10% as bolus, 90% as a one‐hour infusion; maximum dose 90 mg) within three‐hours of the onset of symptoms was shown to improve outcome in carefully selected patients with AIS. Subsequent systematic reviews of all the randomized trials of alteplase involving nearly 7000 patients with AIS have shown the evidence is strong for alteplase to provide an overall net benefit within 4·5 h of onset, despite increased (2–7%) risks of symptomatic intracranial hemorrhage (sICH) and early death. Moreover, the efficacy of the treatment is critically time dependent; earlier use of alteplase provides greater proportional benefit 2, 3.

Compared with the cardiology literature, it is surprising that there has been so little research into the optimal dose of alteplase in AIS, or of the importance of ancillary treatments such as early intensive control of blood pressure (BP), to further improve outcome of such patients. The standard 0·9 mg/kg dose of alteplase was chosen for the NINDS trial on the basis of small pilot dose escalation studies which showed that higher doses were related to the risk of sICH, but no clear correlation was found between treatment and early neurological improvement 4, 5, 6. At about the same time, small double‐blind randomized controlled trials of duteplase (which is similar to rtPA) in patients with AIS in Japan showed that 20 mega‐international units (MIUs) of duteplase (equal to 0·33 MIU/kg or 0·6 mg/kg of rtPA) was not only superior to placebo and comparable with 30 MIU on both angiographic recanalization and clinical improvement, but also that massive ICH was more frequent with 30 MIU of duteplase 7, 8. Low‐dose alteplase (0·6 mg/kg) was subsequently approved in Japan after the Japan Alteplase Clinical Trial 9, an open nonrandomized evaluation of patients within three‐hours of AIS, showed equivalent clinical outcomes and reduced risk of sICH when compared with trials of the standard 0·9 mg/kg dose of alteplase.

Uncertainty over the most efficacious dose and safety of alteplase has resulted in variations in dose regimes and proposed therapeutic response and risks of sICH in the treatment of AIS patients in Asia 10. In many countries outside of Japan, low‐dose alteplase has become an attractive ‘low‐cost’ or ‘safer’ option for those who cannot afford the full dose or are elderly. The high cost of alteplase (∼US$2000 per 2 × 50 mg vials for the 0·9 mg/kg dose) is a major out‐of‐pocket expense for many people in fee‐for‐service health care systems of low‐middle income countries (and even in deprived sections of populations in high income countries) in many parts of the world 11.

There is strong rationale to expect the effects of alteplase to vary across different AIS patient groups and for interaction between dose and hemostatic and cerebrovascular factors. In particular, Asians are more likely to have AIS with lower ‘clot volume’ due to the greater frequency of small vessel or ‘lacunar’ forms of AIS as compared with more large vessel and cardio‐embolic strokes in non‐Asians 12. Standard‐dose alteplase may be better in situations of larger proximal vessel clots, while low‐dose alteplase may be better and safer (i.e. less sICH) for small platelet‐rich clots in more distal or smaller perforating cerebral vessels causing lacunar strokes. Moreover, the Get‐with‐the‐Guidelines stroke quality assurance registry in the United States has shown that Asian AIS patients have an increased risk of sICH with given standard‐dose alteplase 13. As highlighted in a Cochrane review where no clear differences were seen in outcomes through indirect comparisons of different dosages of thrombolytic agents 14, there will be continued uncertainty over the relative benefits and risks of low‐ vs. standard‐dose alteplase in the absence of direct head‐to‐head comparative studies.

As the largest randomized evaluation of alteplase in AIS, the ENhanced Control of Hypertension And Thrombolysis strokE stuDy (ENCHANTED) has been designed as a comparative effectiveness trial to resolve uncertainty over the most efficacious dose of alteplase as well as the most appropriate level of control of elevated BP in the context of AIS. Planning for the study began in 2010 after completion of the pilot phase Intensive Blood Pressure Reduction in Acute Cerebral Hemorrhage Trial (INTERACT1) 15 showed potential benefits of early intensive BP lowering in patients with acute ICH. As low‐dose alteplase and early intensive BP lowering both have the potential to make thrombolytic treatment more efficacious, safer, and affordable in patients with AIS around the world, it was important that ENCHANTED had pragmatic features to allow its efficient conduct in the context of routine clinical practice, and for the results to be widely generalizable.

The study protocol for ENCHANTED has been outlined elsewhere 16. In brief, the study was designed as a quasi‐factorial randomized controlled trial involving two linked comparative treatment arms – ‘alteplase dose’ and ‘BP control’ – with overlapping eligibility criteria. As well as providing flexibility over patient recruitment into one or both treatment arms according to clinician uncertainty and available resources, the study design allows for the two treatment arms to be evaluated separately. The randomization, data collection, follow‐up, and monitoring systems were modeled on the main phase INTERACT2 study 17, where a global network was established to provide a reliable estimate of a clinically worthwhile beneficial effect of a treatment strategy that would influence clinical practice.

Herein we describe the statistical analysis plan (SAP) for the ENCHANTED ‘alteplase dose’ arm, where patient recruitment has been more rapid and the required sample size will be achieved sooner than for the BP control arm. This SAP was completed prior to completion of the data collection, and is what investigators will adhere to in analyzing the results of the study pertaining to the dose of alteplase in AIS. The SAP was approved and signed off by the study Steering Committee in May 2015. Participant recruitment to the alteplase dose arm was completed in August 2015, and final patient follow‐up occurred in November 2015. The statistical analyses specified in the SAP occurred in January 2016, and the main results announced at the European Stroke Organisation conference in Barcelona in May 2016.

Critical appraisal of the noninferiority results of ENCHANTED should follow the principles and criteria used in assessing any study of novel management strategies 18. Special attention will need to be given to assessing the results of both the as‐randomized, intent‐to‐treat, and per‐protocol population analyses, and of any trade‐offs that may arise between the potential benefits (lower risk of sICH) and harms (loss of efficacy in AIS due to large vessel occlusion) of low‐dose compared with standard‐dose alteplase. If the proposed estimates of noninferiority boundary for low‐dose alteplase hold, the results of ENCHANTED could translate into improved effectiveness, safety, and accessibility of thrombolytic treatment for many AIS patients around the world.

## Supporting information


**Appendix S1.** Statistical analysis plan.
