# Supplementary material for: Statistical analysis plan for evaluating low‐ vs. standard‐dose alteplase in the ENhanced Control of Hypertension and Thrombolysis strokE stuDy (ENCHANTED)
Source: Int J Stroke. 2015 Aug 18;10(8):1313–5. doi: 10.1111/ijs.12602 (PMC5324659; doi:10.1111/ijs.12602)
Supplement: Supplementary file 1 — Appendix S1. Statistical analysis plan. [file IJS-10-1313-s001.docx]

**Appendix S1**

**
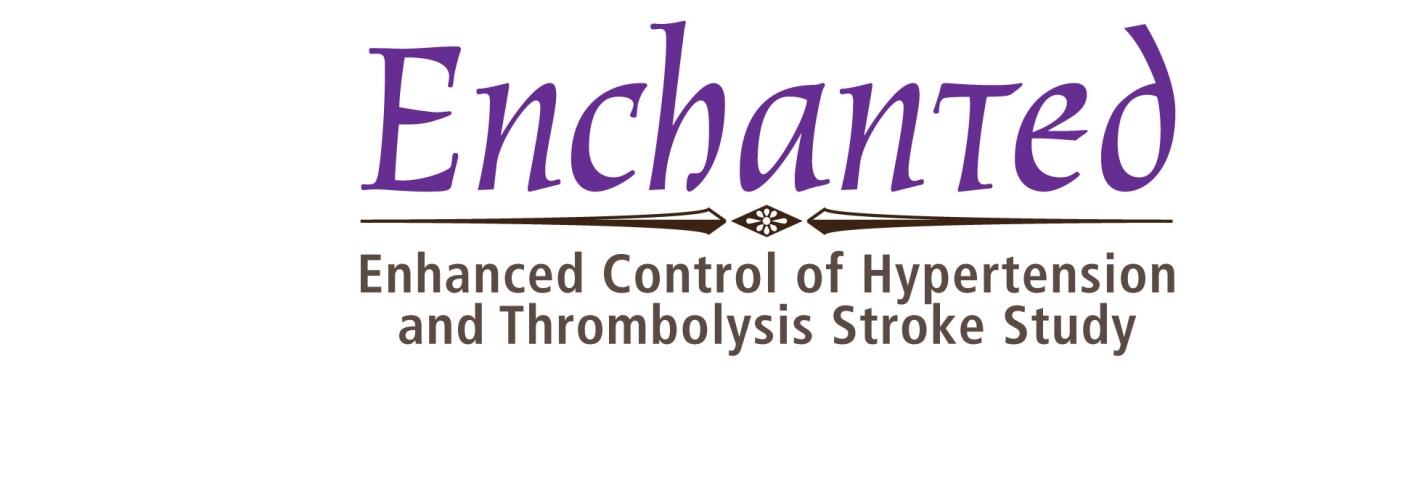
**

**STATISTICAL ANALYSIS PLAN**

***for the***

**alteplase dose arm of this study**

Craig S Anderson,^1^ Mark Woodward,^1^ Hisatomi Arima,^1,2^ Xiaoying Chen,^1^ Richard I Lindley,^1^ Xia Wang,^1^ and John Chalmers;^1^ for the ENCHANTED Investigators

^1^The George Institute for Global Health, Royal Prince Alfred Hospital and University of Sydney

^2^Centre for Epidemiologic Research in Asia, Shiga University of Medical Sciences, Shiga University, Shiga, Japan

**30 May 2015**

**Author for correspondence:**

Professor Craig Anderson

Senior Director, Neurological and Mental Health Division,

The George Institute for Global Health

Royal Prince Alfred Hospital and University of Sydney

Tel: +61 2 9993 4521

Fax: +61 2 9993 4502

Email: canderson@georgeinstitute.org.au

**Contents**

1. List of abbreviations
2. Study objectives
3. Study design
   1. Overview

3.2 Patient population

3.3 Randomization

3.4 Interventions and background care

3.5 Baseline and follow-up assessments

3.6 Assessment of brain imaging

3.7 Sample size and statistical power for alteplase dose arm

3.8 Unblinding

3.9 Definition of outcomes

3.9.1 Primary outcome

3.9.2 Key secondary efficacy outcome

3.9.3 Analysis of efficacy outcomes

3.9.4 Key safety outcome

3.9.5 Other secondary outcomes

3.9.6 Tertiary outcomes

3.9.7 Safety variables

3.9.8 Protocol violations

4. Funding

5. Statistical analysis

5.1 Analysis principles

5.2 Interim analyses

5.3 Dates, vital status and consent-related issues

5.4 Trial profile

5.5 Data sets analyzed

5.6 Patients characteristics and baseline comparisons

5.7 Process measure of background management and treatment

5.8 Alteplase details after randomization

5.9 Primary and key secondary outcomes

5.10 Other secondary outcomes

5.11 Safety endpoints

5.12 Subgroup analysis

5.13 Tables and figures

6. Outline of publication plan

7. References

Appendix 1 Adjudication and classification of intracerebral hemorrhage on brain imaging

Appendix 2 Proposed format of tables for primary publication

Appendix 3 Additional tables

Appendix 4 Proposed content of primary and subsequent publications

# List of abbreviations

| AF | atrial fibrillation |
| --- | --- |
| AHA | American Heart Association |
| AIS | acute ischemic stroke |
| BP | blood pressure |
| CD-ROM | compact disc read-only memory |
| CI | confidence interval |
| CONSORT | consolidated standards of reporting trials |
| CRF/eCRF | case report form / electronic case record form |
| CT | computerized tomography |
| CV | cardiovascular |
| DICOM | digital imaging and communications in medicine |
| DSMB | data safety monitoring board |
| ECG | electrocardiogram |
| ENCHANTED | ENhanced Control of Hypertension And Thrombolysis strokE stuDy |
| FDA | Food and Drug Administration |
| GCP | good clinical practice |
| GCS | Glasgow coma scale |
| HR | heart rate |
| HREC | human research ethics committee |
| HRQoL | health-related quality of life |
| ICC | international coordinating center |
| ICH | intracerebral hemorrhage |
| ICH-GCP | international conference on harmonisation for good clinical practice |
| ie | id est, or that is |
| iqi | interquartile interval |
| IRB | institutional review board |
| ITT | intent-to-treat |
| iv | intravenous |
| IVRS | interactive voice randomisation system |
| LTFU | loss to follow up |
| MedDRA | Medical Dictionary for Regulatory Authorities |
| mins | minutes |
| MIU | mega-international units |
| MRI | magnetic resonance imaging |
| mRS | modified Rankin scale |
| NB | nota bene, or note |
| NHMRC | National Health and Medical Research Council of Australia |
| NIHSS | National Institute of Health stroke scale |
| NINDS | National Institutes of Neurological Diseases and Stroke |
| OC | operations committee |
| OLR | ordinal logistic regression |
| OR | odds ratio |
| PI | principal investigator |
| PP | per-protocol |
| PT | preferred term |
| RCC/RCCs | regional coordinating center / regional coordinating centers |
| rt-PA | recombinant tissue plasminogen activator |
| SAE | serious adverse event |
| SAP | statistical analysis plan |
| SC | steering committee |
| SD | standard deviation |
| SE | standard error |
| sICH | symptomatic intracerebral hemorrhage |
| SOC | system organ class |
| SOP | standard operating procedures |
| TGI | The George Institute for Global Health |
| UK | United Kingdom |
| WHO | World Health Organization |

1. **Study objectives**

The ENCHANTED study involves an assessment of two parallel active-comparison interventional treatment arms – *alteplase dose and intensity of BP control* – on clinical outcomes in patients who fulfil standard eligibility criteria for iv thrombolytic therapy in AIS.

The primary aims of the alteplase dose arm are to evaluate whether:

1. compared with standard-dose (0.9 mg/kg) iv alteplase, low-dose (0.6 mg/kg) iv alteplase has *non-inferior* *efficacy* for the clinical outcome of death or disability at 90 days (the corresponding null hypothesis is that low-dose is inferior to standard-dose alteplase);

*and*

1. compared to standard-dose iv alteplase, low-dose iv alteplase *reduces the harm* of sICH (the corresponding null hypothesis is that there is no difference in the frequency of sICH between the two groups of differing doses of alteplase);

The key secondary efficacy outcome is a shift (‘improvement’) in measures of ‘functioning’ according to the full range of scores on the modified Rankin scale (mRS). Other secondary outcomes include ‘good’ functional outcome on the mRS; separately on death and disability; early neurological deterioration; recurrent acute myocardial infarction and AIS; health-related quality of life (HRQoL); length of hospital stay; need for permanent residential care; and health care costs.

# Study design

**3.1 Overview**

ENCHANTED is an independent, investigator-initiated and conducted, international, multicenter, 2 x 2 quasi-factorial, prospective, open-label, assessor-blinded end-point (PROBE), randomized controlled trial that involves a package of 2 linked comparative treatment arms (‘alteplase dose’ and ‘BP control’). The trial is being conducted in accordance with local and international regulatory and ethical requirements, and ICH-GCP. All participating hospitals received approval from required regulatory authorities, a human research ethics committee (HREC) or an institutional review board (IRB), prior to initiation of the trial. The rationale and design of the study have been described,^1^ and the data will be reported in accordance with Consolidated Standards of Reporting Trials (CONSORT) statements for reporting noninferiority and equivalence randomized trials.^2^ The first patient was randomized in March 2012 and the last patient is expected to be randomized into the alteplase dose arm in August 2015. The trial has been designed so that the treatment arms can be analyzed separately and it is registered with ClinicalTrials.gov (NCT01422616), ISRCTN Register (ISRCTN82387104), and Australian New Zealand Clinical Trial Registry (ACTRN12611000236998).

# 3.2 Patient population

To be eligible, patients with AIS must fulfil local criteria for use of iv alteplase, and the attending investigator clinician is required to sequentially consider their level of clinical uncertainty over the balance of benefits and risks pertaining to arm [A] the appropriate dose of alteplase and arm [B] the level of BP control in each particular patient. Patients will not be eligible if one or more of the following are noted: being unlikely to benefit from alteplase (eg advanced dementia); deemed to have a very high likelihood of death within the next 24 hours; or have another medical illness that is likely to interfere with either the outcome assessments or follow-up. Investigators are able to undertake all investigations according to their usual standard of care in their management of patients with AIS, including urgent referral for cerebral angiography for consideration of endovascular clot retrieval at selected sites. Thus, ENCHANTED is a pragmatic trial designed to evaluate routinely available thrombolytic treatment for AIS in usual best practice conditions.

Before participation, written consent is obtained from each participant or their approved surrogate for patients who are too unwell to comprehend the information. Study investigators may withdraw a patient from the trial at any time without prejudice and explanation. Although study participants/legally acceptable representatives can opt to withdraw at any stage, efforts are always undertaken to obtain outcome data.

**3.3 Randomization**

After confirmation of eligibility, patients are randomized via a central password-protected web-based program developed at The George Institute for Global Health (TGI) in Sydney Australia, to ensure concealment of treatment allocation and to reduce bias. Randomization is done by connecting the study site to the server for registration and randomization of the patient. In China, investigators have the option of using a customised 24 hour digital IVRS that connects to the central server to allow patients to be randomized at sites where rapid access to the internet is not possible.

The randomization sequence uses a minimization algorithm to ensure balance in three key prognostic factors: (i) site of recruitment, (ii) time from the onset of symptoms (<3 versus ≥3 hours) and (iii) National Institutes of Health stroke scale (NIHSS)^3^ score (<10 versus ≥10 points).

Once patient recruitment into Arm A is completed, the randomization system will be re-programmed to allow continued randomization only of patients into Arm B. Fifteen countries are listed in the study: Australia, Brazil, Chile, Columbia, China, Hong Kong, Italy, Korea, Norway, Singapore, Spain, Taiwan, Thailand, United Kingdom, and Vietnam.

**3.4 Interventions and background care**

Site investigators have the choice of randomizing patients into one or both treatment arms of the study: Arm [A] comprises standard-dose (0.9 mg/kg; 10% bolus and 90% infusion over 60 mins; maximum 90 mg) or low-dose (0.6 mg/kg; 15% bolus and 85% infusion over 60 mins; maximum 60 mg) iv alteplase; and Arm [B] comprises intensive BP lowering (target systolic BP 130-140 mmHg within 60-minutes of randomization, and to maintain this level for at least 72 hours, or hospital discharge [or death] if this occurs earlier) or guideline-recommended BP lowering (target SBP <180 mmHg) after the commencement of iv alteplase.

For Arm [A] alteplase dose, the bolus dose has been set to be similar for each treatment group; the only difference between groups is therefore in the total dose of alteplase.

All patients will be managed in a facility with capacity for repeated neurological examination and non-invasive BP and heart rate monitoring (consistent recordings using automatic devices, every 15 minutes for 1 hour, then 6 hourly for 20 hours, then twice daily for 1 week). All BP measurements are from the non-paretic arm (or right arm in situations of coma or tetraparesis), with the patient resting supine for ≥3 minutes. All patients are to receive active care and best practice management according to guidelines, and where neurointervention with intra-arterial thrombolysis and/or endovascular mechanical clot retrieval is allowed, according to local practice.

An acute stroke unit is defined as an area that: is a geographically specific area where patients with acute stroke are managed; has staff organised as part of a coordinated multidisciplinary team; has staff who have special knowledge and skills in the management of acute stroke; provides ongoing education about stroke management for staff, patients and caregivers; and has written protocols for assessment and management of common problems related to stroke.

**3.5 Baseline and follow-up assessments**

Each collaborating hospital site, except those located in the United Kingdom (UK), is required to keep a screening log during the time of participation of all patients presenting with a diagnosis of AIS who are considered for the study but are subsequently excluded. The screening log will record each patient’s initials and date of admission together with a brief description of the main reason as to why he or she was not randomized. The log is used to monitor recruitment and identify specific barriers to randomization of eligible patients. For the UK, all sites contribute to a continuous cycle of national audits, that include the number of stroke admissions, proportion of patients eligible for thrombolysis, and the proportion of patients thrombolyzed, reported on a quarterly basis. This database will be interrogated at the end of the study to assess the proportion of thrombolysis-eligible patients in the UK who are included in ENCHANTED during the study period.

A detailed list of the assessment schedule is contained in the study protocol^1^ and clinical site manuals. Briefly, once informed consent has been obtained, the responsible registered investigator clinician is able to randomize a patient through the secure web-based system after eligibility is confirmed. Data are entered for several key baseline clinical variables including vital signs and scores on the GCS and the NIHSS.^3^ Socio-demographic and clinical history are then recorded on a baseline form and regular checks are made of BP and neurological function over the next 24 and 72 hours, and for 7 days in total, according to a standard protocol. BP is recorded every 15 minutes during the first hour, then hourly for 5 hours, then 6 hourly until 24 hours, and twice daily thereafter for the next 7 days or, should these occur earlier, until death or hospital discharge. Scores on the GCS and NIHSS are recorded at 24 and 72 hours, and at Day 7 (or at hospital discharge). All data on clinical status, treatment and care are recorded prospectively on special prepared worksheets, and subsequently transferred onto eCRFs on the electronic database. All patients are followed daily for 1 week, and then at 28 and 90 days, unless death occurs earlier.

The 28 and 90 day evaluations are conducted in-person or by telephone, by a trained staff member at the local site who is not directly involved with the acute treatment of the subject and is blind to the treatment allocation. In cases where the 90-day assessments are performed by an unblinded assessor, the occurrence will be tallied as a protocol violation and presented in the final study report: nevertheless, the submitted subjects’ data will be used in the analyses.

The hospital coordinator at each collaborating site ensures completion of data. Investigators receive modest reimbursement for their time involved in data collection and for local expenses (eg printing, internet connection, purchase of medications, copying of brain imaging). Patients who do not receive the allocated randomized treatment or do not follow the protocol, are still followed up and analyzed as per the ‘intent-to-treat’ (ITT) principle. Data collection is kept to a minimum to ensure rapid enrolment and follow-up of patients within the context of routine clinical practice.

**3.6 Assessment of brain imaging**

CT scans (or MRIs) are conducted according to standardized techniques at baseline (ie confirmation of diagnosis) in all patients, and at 24-36 hours in as many patients as possible according to usual clinical practice. Uncompressed digital CT images are collected in DICOM format on a CD-ROM identified only with the patient’s unique study number and uploaded by a special purpose-built web-based system for central analysis at TGI. The 24 hour analyzed scans are used to assess for any ICH.

All scans are assessed independently for ICH by at least 2 expert clinical scientists. If classification of the type of ICH is consistent between readers, then the data are recorded directly to the database; if there is inconsistency, a third reader is required to review and finalise the diagnosis and classification of ICH, according to the adjudication procedures outlined in Appendix 1.

The brain imaging system allows assessment of abnormalities using computer-assisted multi-slice planimetric and voxel threshold techniques in MIStar version 3.2 (Apollo Medical Imaging Technology, Melbourne, Victoria, Australia). The system was built to store securely over 10,000 images acquired on participants, with an adjudication system primarily for the recording of ICH. A more sophisticated system is being developed for future analysis of cerebral ischemia using a validated process of recording: (i) *acute ischemic signs* that includes the presence and degree of hypoattenuated tissue (‘mild’ = grey matter attenuation equal to normal white matter; ‘severe’ = grey and white matter attenuation less than normal white matter), acute ischemic lesion extent, ischemic lesion swelling (seven-point scale), presence/absence and location of any hyperattenuated artery; and (ii) *pre-existing (‘brain frailty’) signs* that includes old infarcts (cortical, lacunar, borderzone, brainstem/cerebellar), presence/severity of leukoaraiosis, and cerebral atrophy against standard examples.

**3.7 Sample size and statistical power for alteplase dose arm**

Non-randomized studies suggest that low-dose alteplase provides comparable clinical outcome (ie an odds ratio [OR], lower versus standard dose, of 1.0). For comparison of the treatment effect between low- and standard-dose alteplase, a non-inferiority margin was decided upon that was based on the earlier Cochrane review^4^ which included data from clinical trials before completion of the Third International Stroke Trial (IST-3).^5^ In that review, the OR for death or disability, which occurred in 50% of patients who received alteplase, was 0.76 (95% confidence interval [CI] 0.66-0.87) for alteplase versus control (placebo). Taking a conservative approach, the 40^th^ percentile point (ie the lower limit of 20%CI; exponential (β – 0.26 * SE) around the OR (0.77) rather than the observed OR was taken as a more robust reference to describe the effects of standard-dose alteplase. This translates into a margin of excess risk of placebo versus standard-dose alteplase of 1.29. According to standard approaches,^6^ a non-inferiority margin of 1.14 has been set to provide assurance that low-dose alteplase retains at least half the efficacy of standard-dose alteplase. Provided that the upper limit of 95% CI of low- versus standard-dose alteplase is less than this non-inferiority margin, we shall conclude there is non-inferiority. The sample size decided upon takes account of the potential for a negative interaction between intensive BP lowering and low-dose alteplase. That is, the primary event rates were estimated at 46.25% in those receiving standard-dose rtPA and 46.75% in those receiving low-dose rtPA, such that the absolute non-inferiority margin rate will be 6.5%. Resulting calculations produced a sample size of 3300 (1650 per group) to provide at least 90% power (1-sided α=0.025) for achieving the non-inferiority setting, assuming 5% drop outs due to missing data from loss-to-follow-up (LTFU) and protocol violations. Provided non-inferiority is confirmed, we shall proceed to evaluate if low-dose alteplase is superior to standard-dose alteplase.

The most recent Cochrane review of thrombolysis in AIS notes the rate of death or disability (mRS score of 2-6) in patients treated with alteplase as 64%.^7,8^ This review included data from IST-3, which was undertaken specifically to assess the effectiveness of alteplase in patients who do not exactly meet the license criteria for the treatment, including in the very old (>80 years) and for use out to 6 hours after the onset of symptoms (33% [1009/3035] of participants were included beyond 4.5 hours from stroke onset). The review indicates that the odds of a poor outcome (mRS 2-6) was 0.79 (95%CI 0.71-0.88), but there was significant heterogeneity between trials. In a meta-analysis of individual patient data from randomized trials,^9^ alteplase was associated with reduced poor outcome within 3 hours (OR 0.57, 95%CI 0.44-0.74) and between 3-4.5 hours (OR 0.79, 95%CI 0.66-0.95) but not after 4.5 hours (OR 0.87, 95%CI 0.71-1.05). Thus, we have decided to maintain the planned non-inferiority margin of 1.14 with the intent that most participants will be randomized to alteplase within 3 hours after the onset of symptoms.

Based on the results of past epidemiological studies^10,11^ and assuming a potential interaction between low-dose alteplase and intensive BP lowering where both have the potential to reduce the risk of sICH, a sample size of 3300 (1650 patients per group) will also provide >80% power (2-sided α=0.05) to detect a >40% relative reduction in the risk of sICH for the low-dose compared to standard-dose alteplase, assuming that 5% of patients drop out during follow-up.

**3.8 Unblinding**

In accordance with standard operating procedures (SOP) of TGI, the number of people having access to the interim data/results is kept to a minimum, and only includes members of the Data and Safety Monitoring Board (DSMB) and associated statisticians responsible for writing the reports. Statisticians not involved in the writing of the DSMB reports will remain blinded until the final study results are released, and will work on dummy datasets to develop, and test, the statistical computer code. Treatment allocations are stored securely in a separate location to other data for this purpose.

The SAP was written by the principal investigator, two independent statisticians, and several other investigators, who will be kept blind to the treatment allocations and study results until the final study results are released.

# 3.9 Definition of outcomes

*3.9.1 Primary outcome*

The primary outcome is the occurrence of a poor outcome at 90 days after randomization. This outcome is measured using the mRS,^12,13^ the most widely used instrument for grading the impact of stroke treatments, that assesses daily functioning through the categorization of levels of disability (or ‘dependency’, sometimes equated to ‘handicap’). The broad mRS scaling is: 0 = no symptoms at all; 1 = no significant disability despite symptoms, but able to carry out all usual duties and activities; 2 = slight disability, unable to carry out all previous activities but able to look after own affairs without assistance; 3 = moderate disability requiring some help, but able to walk without assistance; 4 = moderate-severe disability, unable to walk without assistance and unable to attend to own bodily needs without assistance; 5 = severe disability, bedridden incontinent, and requiring constant nursing care and attention; 6 = dead. The protocol states that scores of 2–6 (ie death or disability) are defined as a poor outcome and scores of 0-1 as an excellent outcome. Scores of 0-2 is defined as a good outcome.

*3.9.2 Key secondary efficacy outcome*

The key secondary outcome is shift (‘improvement’) in measures of functioning according to the full range of scores on the mRS at 90 days.^14-16^ Although ordinal approaches to analysis of the mRS are increasingly being used to appreciate the effects of medical^5,17^ and endovascular reperfusion therapies^18-20^ in patients with AIS and to improve statistical efficacy, we chose to keep this as a secondary endpoint as the trial aims to challenge the regulatory approved dose of alteplase which was based on conventional dichotomous analysis of the mRS in the National Institute of Neurological Disorders and Stroke (NINDS) trial.^21^

*3.9.3 Analysis of efficacy outcomes*

The primary analysis of the mRS will be unadjusted and undertaken using the conventional dichotomous (i.e. 0-1 versus 2-6) separation of the mRS. A sensitivity analysis will also be undertaken, using logistic regression, with adjustment for the minimization variables, including NIHSS as a continuous variable, and several other prognostic covariates: age, sex, ethnicity, pre-morbid mRS (0 or 1), pre-morbid use of aspirin, other antiplatelet agent or warfarin, and any history of stroke, coronary artery disease, diabetes mellitus, and atrial fibrillation (AF).

The primary analysis of the treatment effect on the key secondary outcome (a ‘shift’ on the mRS) will also be unadjusted and analysed using ordinal logistic regression (OLR). OLR assumes proportionality of odds, which will be tested using a likelihood ratio test. The effect of low versus high dose alteplase will be summarised by an OR and 95% CI. The Howard method^16^ will be used as a sensitivity analysis to describe the distribution of categories on the mRS. A further sensitivity analysis will be undertaken using the same covariates as outlined above, in an adjusted analysis.

*3.9.4 Key safety outcome*

The key safety outcome is sICH, defined according to the Safe Implementation of Thrombolysis in Stroke-Monitoring Study (SITS-MOST),^22^ as large local or remote parenchymal ICH (type 2, defined as greater than 30% of the infarcted area affected by hemorrhage with mass effect or extension outside the infarct) combined with neurological deterioration (≥4 points on the NIHSS) or leading to death within 24-36 hours.

Other definitions of sICH will be used that include:

- the NINDS trial^21^ criteria of any ICH associated with neurological deterioration (≥1 point change in NIHSS score) from baseline or death within 24-36 hours;
- the European-Australian Cooperative Acute Stroke Study 2 (ECASS2)^23^ of any ICH with neurological deterioration (≥4 points on the NIHSS) from baseline or death within 24-36 hours;
- clinician-reported ICH as a serious adverse event (SAE);
- fatal ICH, defined by any parenchymal ICH of type 2 and death within 7 days.

*3.9.5 Other secondary outcomes*

Other secondary outcomes will comprise the following.

- ICH of any type on brain imaging ≤7 days of treatment
- Cause-specific mortality within the 90-day follow-up period. The primary cause of death will be categorised as:
  - death from direct effects of the initial AIS (within 7 days unless there is a definite alternative cause);
  - death from pneumonia or other complications of the AIS (beyond 7 days unless there is a definite alternative cause;
  - death from a serious cardiovascular (CV) event other than acute stroke;
  - death from recurrent acute stroke;
  - Death or major disability, according to scores 3-6 on the mRS
  - Death or neurological deterioration (≥4-points decline in NIHSS) within 24 hours,
  - Duration of initial hospitalisation in days
  - Health-related quality of life (HRQoL), as assessed on the EuroQoL,^24^ as an overall health utility score (ED-5D-3L) at 90 days

*3.9.6 Tertiary outcomes*

Tertiary outcomes will include the following:

- All-cause mortality at 7 and 28 days
- Place of death (in initial acute hospital, another hospital or institutional facility, at home)
- Trends in physical functioning on the mRS over 7, 28 and 90 days
- Duration of stay in an intensive care unit
- Use of endovascular clot retrieval therapy
- Separate components of the EQ-5D - mobility, self-care, usual activities, pain/discomfort, and anxiety/depression - at Days 28 and 90.

# *3.9.7 Safety variables*

All deaths and ICH are adjudicated by a blinded central expert committee. Since all patients have AIS, deaths are classified as being due to this condition within 7 days, unless an unequivocal non-cerebral cause was established. SAEs are reported according to standard definitions and coded using terminology of the Medical Dictionary for Regulatory Authorities (MedDRA). However, as this a classification by System-Organ Class (SOC) and Preferred Term (PF) that are not necessarily relevant for this study, the following categories of SAEs derived from MedDRA are defined:

- Neurological deterioration as a direct effect of the AIS with or without evidence of mass effect or ICH on repeat brain imaging;
- Major extracranial hemorrhage;
- Recurrent AIS with the onset of focal neurological symptoms and signs consistent with acute stroke occurring more than 24 hours after the primary event without alternative diagnosis explained on repeat brain imaging;
- Recurrent AIS with neurological symptoms and signs consistent with acute stroke and brain imaging has excluded ICH and non-stroke lesion;
- Recurrent acute stroke syndrome without confirmatory brain imaging or necropsy;
- Acute coronary event according to standard definitions consistent with a typical clinical presentation, abnormal electrocardiogram, or abnormally elevated enzymes;
- Other CV event including sudden death.

SAEs will be further categorised into fatal and non-fatal SAEs with a similar structure.

*3.9.8 Protocol violations*

Patients who have one or more of the following protocol violations will be excluded from the per-protocol (PP) population: age <18 years; final diagnosis not AIS; systolic BP >185 mmHg (inclusion criteria BP level); randomized >4.5 hours; final diagnosis unknown/uncertain because of missing source documents or neuroimaging; failure to receive alteplase at either the correct bolus or infusion dose, and failure to obtain a blind assessment of the 90-day outcome. The range of alteplase doses considered ‘low’ or ‘standard’ are those above and below the mid-point between study doses, that is defined as <0.75 versus ≥0.75 mg/kg, respectively. For the PP analysis, the dose of alteplase is based primarily on measured body weight, either pre-randomisation or after admission to hospital; if these data are missing, estimated body weight will be used to define the dose of alteplase. Definitions for a protocol violation on alteplase dose are: low-dose outside 0.6-0.75 mg/kg range and standard-dose outside of 0.75-0.9mg/kg range.

1. **Funding**

The main source of funding for the study is from the National Health and Medical Research Council (NHMRC) of Australia (Project Grant number 1020462). Other sources of funding are from the Stroke Association of the UK (Reference TSA 2012/01), the National Council for Scientific and Technological Development of Brazil (CNPq grant number 467322/2014-7), and the Ministry for Health, Welfare and Family Affairs of the Republic of Korea (HI14C1985). The sponsors of the study had no role in study design, data collection, data analysis, data interpretation, or writing of the report. The corresponding author had full access to all the data in the study and had final responsibility of the decision to submit the SAP for publication.

**5. Statistical analysis**

# 5.1 Analysis principles

- Analyses will be conducted on an ITT basis.
- A sensitivity PP analysis will also be carried out on patients who fulfil inclusion criteria with definite AIS who actually received the correct randomized dose of alteplase within 4.5 hours of the onset of symptoms and had a 90-day blind outcome assessment.
- Analysis of the primary and key secondary efficacy endpoints will be based on a non-inferiority margin of 1.14. The upper limit of 95% CI of low- versus standard-dose alteplase should be less than this margin, at a one-side α level of 0.025, to provide assurance that low-dose alteplase retains at least half the efficacy of standard-dose alteplase. If non-inferiority is verified, low-dose alteplase will be tested for superiority using a two-tailed α significance level of 0.025.
- Analysis of the safety endpoint of sICH will be for superiority of low- versus standard-dose alteplase. All tests are two-sided and the nominal level of α will be 5%.
- The primary analysis of the treatment effects will be unadjusted. Analysis of the treatment effects on the primary and key secondary outcomes will have a sensitivity analysis with adjustment for the minimization and key prognostic covariates of age, sex, ethnicity, pre-morbid mRS, pre-morbid use of aspirin or other antiplatelet agent, and history of stroke, coronary artery disease, diabetes mellitus and AF.
- Adjusted analyses will also include multiple imputation by chained equations with 30 imputations should the number of missing observations be substantial (ie >10%). The number of observations used in such analysis will be reported. Last observations will not be carried forward unless this is necessary.
- Subgroup analyses will be carried out irrespective of whether there is a significant treatment effect on the primary outcome. These analyses will be unadjusted.
- No formal adjustments will be undertaken to constrain the overall type I error associated with the secondary, tertiary and exploratory analyses. Their purpose is to supplement evidence of the primary analysis of the treatment effect. P values will therefore not be adjusted for multiplicity. However, the outcomes are clearly categorized by degree of importance (primary to tertiary) and a limited number of subgroup analyses will be pre-specified. Results will be interpreted in this context.
- Analyses will be conducted primarily using SAS software.

# 5.2 Interim analyses

An independent DSMB, chaired by Professor John Simes of the Clinical Trials Centre at the University of Sydney, and consisting of clinicians and biostatisticians, reviewed unblinded data from the ENCHANTED study at twice-yearly intervals during conduct of the trial. The DMSB Charter outlined the need to review recruitment, BP separation, dropout and event rates, monitor safety endpoints, and examine the effect of treatment on efficacy outcomes. They were also charged with informing the study Steering and Operational Committees if at any time there emerges either evidence beyond reasonable doubt of a difference between randomized groups in the primary outcome, or evidence that is likely to change clinical practice in the context of current knowledge.

Two formal interim analyses after approximately 30% and 60% of the patients had been followed-up for 30 days were planned and actually conducted. The Haybittle-Peto stopping rule was used (ie a difference 3 SE is considered to be clear evidence of a treatment effect). The study was not terminated early and no additional looks to the discretion of the DSMB were added. The final last level of significance with 3 looks will therefore be 0.0482. Although naïve estimates can theoretically be slightly biased when a stopping rule is used, there will be no correction of the estimates on termination as the bias is likely to be negligible with this design.

**5.3 Dates, vital status and consent-related issues**

The study is conducted at sites with experience in acute stroke care. Regionally-based experienced clinical research monitors perform online and on-site data verification; site monitoring is undertaken, initially after the first few patients were randomized at sites, and thereafter the frequency of monitoring was determined by patient recruitment numbers and data quality whilst site staff continued to participate in the trial. As this is an open trial of differing management strategies in a critical illness, monitoring serves to confirm that investigators are adhering to the protocol and ICH-GCP guidelines, and the accuracy of the data. Site monitoring aims to confirm: (i) demographic and consent details on all randomized patients; (ii) details of all SAEs against source documents; (iii) collect/correct any outstanding/missing data; and (iv) check selected variables against source medical documents in approximately 10% random selection of patients. Key data points, vital status at final follow-up, dates and details of any deaths are queried in order for no missing values to remain at the end of the study.

Two important situations can lead to the cessation of participation in the study: a patient, next of kin or legal surrogate may withdraw consent; or they may refuse continuation of the study treatment when delayed consent is sought. For both situations, the study treatment will cease and the patient will receive appropriate treatment as determined by the attending clinician. The information statement provided to the patient and/or the next of kin or surrogate clearly states that the patient can be withdrawn from the study at any time without prejudice and explanation. Such withdrawal is documented in the patient’s file. If withdrawal of consent relates to the study treatment alone, data collection can continue on documentation of this fact in the patient’s files. If consent for use of data is withheld, the patient’s data will be removed from the analysis, except for data related to consent. Censoring dates will be used only in cases of ‘real’ LTFU, such that the date of censoring will be the last day of contact, or the date of hospital discharge, if not other information is available.

In cases where the 90-day assessments were performed by an unblinded assessor, as assessed by requesting knowledge of the assessor at follow-up, such occurrences will be tallied as protocol violation and presented in the final study report: nevertheless, the submitted subjects’ data will be used in the analysis.

**5.4 Trial profile**

Flow of patients through the study will be displayed in a CONSORT diagram, shown in the Appendix 2 (figure 1). The report will include: the number of screened patients who met study inclusion criteria and the number of patients who are included; and reasons for exclusion of non-included patients and accompanying information. In addition, the number of patients randomized outside the time window and other protocol deviations will be provided, as outlined in Appendix 3 (table 1).

**5.5 Data sets analyzed**

- ITT population - the data set of *all patients randomized* in the study without exclusion and the analysis conducted according to the ITT principle. This will be used to assess both efficacy and safety.
- Analyzed data set - includes data from all randomized patients in the study who are known to have died or with mRS scores at 90 days.
- PP population – the data set includes all randomized participants with the primary outcome recorded and who did not have a relevant protocol violation.

# 5.6 Patients characteristics and baseline comparisons

Description of the following baseline characteristics will be presented by treatment group as outlined in Appendix 2 (table 1). Discrete variables will be summarised by frequencies and percentages. Percentages will be calculated according to the number of patients in whom data are available. If missing values are important, the denominator will be added in a footnote in the corresponding summary table. In some instances, frequencies and percentage of patients in subcategories of variables will be reported, for example by age (10-year strata), region (by country of recruitment) and time from stroke onset to randomizaton (1 hour strata) (Appendix 3, table 2).

Continuous variables will be summarised by use of standard measures of central tendency and dispersion, either mean and SD for variables identified with #, or median and interquartile interval (iqi) with ^†^. Durations will also be summarised by medians and iqi.

Baseline measures for all patients will be tabulated for the following variables: age #, sex, ethnicity (Asian vs other), geographical region (China, other Asia, Australia/Europe, and South America), systolic BP #, diastolic BP #, heart rate #, NIHSS score ^†^, GCS score ^†^, medical history (prior stroke, hypertension, coronary heart disease, AF, diabetes mellitus, and smoking status), time between onset and randomization ^†^, medications at time of admission, final diagnosis of presumed pathological type of AIS, and presence of cerebral infarction.

**5.7 Process measures of background management and treatment**

This will be described as outlined in Appendix 2 (table 2). Counts and percentages will be calculated per treatment arm for all items of standard stroke care. The period covers Day 0 (randomization) to Day 7. A P value from a χ^2^ test or Fisher test will also be reported. The default analysis is based on the χ^2^ test unless any expected number per cell is <5, in which case a Fisher test will be used.

# 5.8 Alteplase details after randomization

These will also be outlined in Appendix 2 (table 2). Details of the weight of patients used to calculate the dose of alteplase, and the time to treatment, and bolus and infusion doses will be summarised by treatment arm. Counts and percentages will be displayed for all categorical items. Continuous outcomes will be summarised by either means (SD) or medians (iqi) as further detailed in the same table.

# 5.9 Primary and key secondary outcomes

The primary analysis of the effect of treatment on the primary measure of poor outcome will be undertaken using the traditional dichotomous (i.e. 0-1 versus 2-6) separation of the mRS, as outlined in Appendix 2 (table 3). A standard χ^2^ test will be used as the primary test of statistical significance on the effect of treatment allocation on poor outcome. Frequencies and % per arm, and an OR measuring the treatment effect and its 95% CI will also be reported. We will also perform adjusted analyses for sensitivity purposes. They will be based on a multivariable logistic regression analysis adjusted for randomization strata and key prognostic covariates: age, sex, ethnicity, pre-morbid mRS (0 or 1), pre-morbid use of aspirin, other antiplatelet agent or warfarin, and any history of stroke, coronary artery disease, diabetes mellitus, and AF. If the missing data exceed 10%, a sensitivity analysis based on multiple imputations will be performed to see how this affects the outcome. If there are notably different conclusions from the analysis based on approaches to analysis of the mRS, the reasons for such differences will be explored in secondary analysis to be published after the primary paper. The primary analysis of the key secondary outcome will be based on unadjusted ordinal logistic regression across all levels of the mRS at 90 days. This analysis assumes a common OR across all cut points of the mRS. A shift figure will be presented to illustrate the change distribution across treatment arm. If the proportional-odds assumption is violated in either of these analysis (i.e. significant P value for the Brant test of common OR) a model with non-proportional odds will be fitted.

# 5.10 Other secondary outcomes

All binary secondary outcomes will be analysed by means of a χ^2^ test, except that a Fisher test will be used if any expected numbers are <5. These data will be summarised by an OR and its 95% CI as before. The effect of treatment on survival time or any time-to-event type of outcome will be tested by means of a log-rank test. Continuous endpoints, such as the health utility score (ED-5D) at 90 days, will all be summarised by medians (iqi). A difference between medians and its 95% CI might be computed if this is feasible and required in subsequent publications. Probability of survival by treatment group may be presented as Kaplan-Meier curves. Length of stay in hospital and in an intensive care unit will be censored due to early deaths or stays longer than 90-days (Appendix 2, table 4); they will therefore be considered as times to discharge and analyzed with a log-rank test.

# 5.11 Safety endpoints

Counts and percentages per treatment arm will generally summarise all specific pre-defined SAE categories, as outlined in Appendix 2 (table 5). These generally represent the number of patients experiencing a specific SAE (at least once), fatal ones, and the breakdown by subcategory (when appropriate). This includes evidence of an early neurological deterioration within 24-36 hours, the various forms of vascular and non-vascular events (i.e. pneumonia, sepsis, and fracture). A global chi-square (or Fisher test, if any expected value is <5) of a treatment effect will be carried out and its P value reported. A measure of treatment effect (i.e. OR and its 95% CI) might be reported if appropriate. None of the above analyses will be adjusted. A similar breakdown of the SAEs stratified by fatal/nonfatal status will also be presented. In addition, a table displaying all-cause mortality after the first 7 days and the primary cause of death (globally and per category) may be provided. All deaths occurring during the first week will be considered as stroke deaths unless otherwise specified. The same rules for the tests, OR and 95% CI apply.

# 5.12 Subgroup analysis

Analysis of 10 key subgroups will be carried out of the primary outcome, outlined in Appendix 2 (table 5). Unadjusted p-values will be reported. The pre-specified subgroup analyses are as follows:

1. Age: <65 versus ≥65 years
2. Sex: male versus female
3. Ethnicity: ‘Asian’ (i.e. Chinese or other Asian) versus ‘Non-Asian’ (i.e. by groups defined as African, Arabic, Australian, Caucasian/European, Indian subcontinent, Maori/Polynesian, Mixed, and Latin American)
4. Time to from symptom onset to randomisation: <3 versus ≥3 hours
5. Systolic BP at baseline: below and above mean
6. Baseline NIHSS score: above and below overall median
7. Final diagnosis of AIS at the time of hospital separation:
   - Large artery occlusion/stenosis on extra– or intra-cranial atheroma
   - Small vessel or perforating vessel ‘lacunar’ disease
   - Cardio-embolic
   - Other definite or uncertain pathological diagnosis
8. Presence of AF based on definite history of AF or current AF confirmed on ECG at presentation: yes/no
9. (Investigator reported) presence of cerebral infarction on baseline CT scan: yes/no
10. Pre-morbid use of aspirin or other antiplatelet therapy: yes/no

The main analysis for each subgroup will be an interaction test in a logistic regression model to determine whether the effect of treatment differs significantly across categories for that particular subgroup. Summary measures could include counts, percentages and a measure of effect size (OR) with its 95% CI obtained from a stratified analysis.

For each treatment by subgroup interaction, the change in log likelihood when the interaction term is added to a logistic regression model containing the treatment and subgroup main effects will be calculated. The significance of the interaction will be assessed by comparing the change in log likelihood with percentage points of a χ^2^ distribution with the appropriate degrees of freedom (a likelihood ratio test). Where a factor has more than two levels, the test is for the null hypothesis that all levels have the same underlying OR versus the alternative that the OR have a linear trend (if the levels are ordered), or simply that the OR are not all equal (if the levels are not ordered). The cut points for continuous variables have been chosen by reference to an analysis of baseline characteristics (both treatment groups combined) so as to maximize power.

Subgroup results will be presented as forest plots, with P values for heterogeneity for each pair of subgroups.

# 5.13 Tables and figures

These are outlined in Appendix 2. Table 1 will report all collected baseline characteristics of the participants by treatment group. Table 2 will report on the use of alteplase according to estimated or direct measurement of body weight: number of patients treated with alteplase, bolus and infusion dosage, and the times from randomization and stroke onset to treatment. This table will also report on process measures, concomitant treatments and details of management. Table 3 will display the results for primary and secondary outcomes. Table 4 will present other secondary outcome, and selected relevant tertiary outcomes. Table 5 will report SAEs to the end of follow-up. Subgroup analyses are presented in Table 5 and with a forest plot.

In addition, the following figures will be prepared:

- A CONSORT diagram illustrating the flow of patients through the study (figure 1).
- A bar chart displaying each grade on the mRS in each treatment group (figure 2).
- A plot of non-inferiority treatment effects on the primary outcome according to the primary analysis, and for the sensitivity and PP analyses (figure 3).
- A forest plot of the treatment effects on the primary outcome according to the pre-specified subgroups. (Figure 4).
- Depending on the primary results, a plot may be included that displays the best linear fit between the log OR for a excellent stroke outcome (mRS 0-1) for patients given low-dose alteplase compared with those given standard-dose alteplase and treatment delay (in hours). Estimates will be derived from a regression model in which alteplase dose, age, and stroke severity are included as main effects with a treatment x time interaction variable (figure 5).

**6. Outline of publication plan**

Appendix 4 outlines the proposed papers and their anticipated timelines for analysis of the ENCHANTED alteplase dose arm of the study.

1. **References**
2. Huang Y, Sharma VK, Robinson T, et al; the ENCHANTED investigators. Rationale, design, and progress of the ENhanced Control of Hypertension And Thrombolysis strokE stuDy (ENCHANTED) trial: an international multicenter 2 × 2 quasi-factorial randomized controlled trial of low- vs. standard-dose rt-PA and early intensive vs. guideline-recommended blood pressure lowering in patients with acute ischaemic stroke eligible for thrombolysis treatment. *Int J Stroke* 2015; 2 APR 2015, DOI: 10.1111/ijs.12486
3. Piaggio G, Elbourne DR, Pocock SJ, Evans SJW, Altman DG; for the CONSORT Group. Reporting of noninfeririority and equivalence randomized trials: extension of the CONSORT 2010 statement. *JAMA* 2012;308:2594-2604.
4. Brott T, Adams HP, Jr., Olinger CP, et al. Measurements of acute cerebral infarction: a clinical examination scale. *Stroke* 1989; 20: 864-870.
5. Wardlow JM, Murray V, Berge E, del Zoppo GJ. Thrombolysis for acute ischaemic stroke. *Cochrane Database of Systematic Reviews 2009*, Issue 4, Art. No.:CD000213. DOI: 10.1002/14651858.CD000213.pub2
6. Sandercock P, Wardlaw JM, Lindley RI, et al; the IST-3 collaborative group. The benefits and harms of intravenous thrombolysis with recombinant tissue plasminogen activator within 6 h of acute ischaemic stroke (the third international stroke trial [IST-3]): a randomised controlled trial. *Lancet* 2012;379:2352-63.
7. Food and Drug Administration. Guidance for industry: non-inferiority clinical trials 2010 (http://www.fda.gov/downloads/drugs/guidance/ucm202140.pdf).
8. Wardlaw JM, Murray V, Berge E, del Zoppo G, Sandercock P, Lindley RL, Cohen G. Recombinant tissue plasminogen activator for acute ischaemic stroke: an updated systematic review and meta-analysis. *Lancet* 2012; 379:2364-2372.
9. Wardlaw JM, Murray V, Berge E, del Zoppo GJ. Thrombolysis for acute ischaemic stroke. *Cochrane Database of Systematic. Reviews 2014*, Issue 7. Art. No.: CD000213. DOI: 10.1002/14651858.CD000213.pub3.
10. Emberson J, Lees KR, Lyden P, et al; for the Stroke Thrombolysis Trialists’ Collaborative Group. Effect of treatment delay, age and stroke severity on the effects of intravenous thrombolysis with alteplase for acute ischaemic stroke: a meta-analysis of individual patient data from randomised trials. *Lancet* 2014; 384; 1929-1935.
11. Tsivgoulis G, Frey JL, Flaster M, et al. Pre-tissue plasminogen activator blood pressure levels and risk of symptomatic intracerebral hemorrhage. *Stroke* 2009;40:3631-3634.
12. Wahlgren N, Ahmed N, Eriksson N, et al. Multivariable analysis of outcome predictors and adjustment of main outcome results to baseline data profile in randomized controlled trials: Safe Implementation of Thrombolysis in Stroke-MOnitoring STudy (SITS-MOST). *Stroke* 2008;39:3316-3322
13. Banks JL, Marotta CA. Outcomes validity and reliability of the modified Rankin scale: implications for stroke clinical trials: a literature review and synthesis. *Stroke* 2007; 38: 1091-1096.
14. Quinn TJ, Dawson J, Walters MR, Lees KR. Reliability of the modified Rankin Scale: a systematic review. *Stroke* 2009; 40: 3393-3395.
15. Saver JL. Novel end pont analytic techniques and interpreting shifts across the entire range of outcome scales in acute stroke trials. *Stroke* 2007; 38: 3055-3062.
16. Bath PMW, Lees KR, Schellinger PD, Altman H, Bland M, Hogg C, Howard G, Saver JL. Statistical analysis of the primary outcome in acute stroke trials. *Stroke* 2012; 43: 1171-1178.
17. Howard G, Waller JL, Voeks JH, Howard VJ, Jauch EC, Lees KR, Nichols FT, Rahlfs VW, Hess DC. A simple, assumption-free, and clinical interpretable approach for analysis of modified Rankin outcomes. *Stroke* 2012; 43: 664-669.
18. Bath P, Woodhouse L, Scutt P, et al; the ENOS Trial Investigators. Efficacy of nitric oxide, with or without continuing antihypertensive treatment, for management of high blood pressure in acute stroke (ENOS): a partial-factorial randomised controlled trial. *Lancet* 2014;385:617-628
19. Berkhemer O, Fransen P, Beumer D, et al; the MR CLEAN Investigators. A randomized trial of intraarterial treatment for acute ischemic stroke. *New Engl J Med* 2015;372:11-20.
20. Campbell B, Mitchell P, Kleinig T, et al; the EXTEND-IA Investigators. Endovascular therapy for ischemic stroke with perfusion-imagine selection. *New Engl J Med* 2015;372:1109-18.
21. Goyal M, Demchuk A, Menon B, et al; the ESCAPE Trial Investigators. Randomized assessment of rapid endovascular treatment of ischemic stroke. *New Engl J Med* 2015;372:1019-30.
22. The National Institute of Neurological Disorders and Stroke rt-PA Stroke Study Group: tissue plasminogen activator for acute ischemic stroke. *New Engl J Med* 1995;333:1581-1587.
23. Wahlgren N, Ahmed N, Dávalos A, Ford GA, Grond M, Hacke W, et al; SITS-MOST investigators. Thrombolysis with alteplase for acute ischaemic stroke in the Safe Implementation of Thrombolysis in Stroke- Monitoring Study (SITS-MOST): an observational study. *Lancet* 2007;369:275–282.
24. [Hacke W](http://www.ncbi.nlm.nih.gov/pubmed/?term=Hacke%20W%5BAuthor%5D&cauthor=true&cauthor_uid=9788453), [Kaste M](http://www.ncbi.nlm.nih.gov/pubmed/?term=Kaste%20M%5BAuthor%5D&cauthor=true&cauthor_uid=9788453), [Fieschi C](http://www.ncbi.nlm.nih.gov/pubmed/?term=Fieschi%20C%5BAuthor%5D&cauthor=true&cauthor_uid=9788453), [von Kummer R](http://www.ncbi.nlm.nih.gov/pubmed/?term=von%20Kummer%20R%5BAuthor%5D&cauthor=true&cauthor_uid=9788453), [Davalos A](http://www.ncbi.nlm.nih.gov/pubmed/?term=Davalos%20A%5BAuthor%5D&cauthor=true&cauthor_uid=9788453), [Meier D](http://www.ncbi.nlm.nih.gov/pubmed/?term=Meier%20D%5BAuthor%5D&cauthor=true&cauthor_uid=9788453), [Larrue V](http://www.ncbi.nlm.nih.gov/pubmed/?term=Larrue%20V%5BAuthor%5D&cauthor=true&cauthor_uid=9788453), [Bluhmki E](http://www.ncbi.nlm.nih.gov/pubmed/?term=Bluhmki%20E%5BAuthor%5D&cauthor=true&cauthor_uid=9788453), [Davis S](http://www.ncbi.nlm.nih.gov/pubmed/?term=Davis%20S%5BAuthor%5D&cauthor=true&cauthor_uid=9788453), [Donnan G](http://www.ncbi.nlm.nih.gov/pubmed/?term=Donnan%20G%5BAuthor%5D&cauthor=true&cauthor_uid=9788453), [Schneider D](http://www.ncbi.nlm.nih.gov/pubmed/?term=Schneider%20D%5BAuthor%5D&cauthor=true&cauthor_uid=9788453), [Diez-Tejedor E](http://www.ncbi.nlm.nih.gov/pubmed/?term=Diez-Tejedor%20E%5BAuthor%5D&cauthor=true&cauthor_uid=9788453), [Trouillas P](http://www.ncbi.nlm.nih.gov/pubmed/?term=Trouillas%20P%5BAuthor%5D&cauthor=true&cauthor_uid=9788453). Randomised double-blind placebo-controlled trial of thrombolytic therapy with intravenous alteplase in acute ischaemic stroke (ECASS II). Second European-Australasian Acute Stroke Study Investigators. [*Lancet*](http://www.ncbi.nlm.nih.gov/pubmed/9788453) 1998;352:1245-1251.
25. Rabin R, de Charro F. EQ-5D: a measure of health status from the EuroQol Group. *Ann Med* 2001; 33: 337-343.

**Appendix 1**

**Adjudication and classification of intracerebral hemorrhage on brain imaging**

**Clinician scientists provide responses to the following questions:**

1. Is there any evidence of hemorrhage on this scan? Yes/No

If No, proceed to question 3

If Yes, code bleeding as follows:

- 1. HI1 Small petechiae along the margins of the infarct Yes/No
  2. HI2 Confluent petechiae within infarcted area but no space occupying effect Yes/No
  3. PH1 Blood clots in <30% of infarcted area with slight space-occupying effect Yes/No
  4. PH2 Blood clots in >30% of infarcted area with substantial space-occupying effect Yes/No

And respond to following:

1. Subarachnoid Yes/No
2. Intraventricular Yes/No
3. Subdural Yes/No
4. Other Yes/No
5. In your opinion, will this hemorrhage have been the predominant cause of the neurological worsening? Yes/No
6. Assessment of swelling.

Is there any evidence of midline shift Yes/No

Abbreviations: HI denotes hemorrhagic infarction; PH, parenchymal hemorrhage

**Appendix 2**

**Proposed format of data tables and figures for main results publication**

**Table 1: Baseline characteristics**

|  | Low-dose group  (n=xxx) | Standard-dose group  (n=xxx) |
| --- | --- | --- |
| **Time from stroke onset to randomization** (hrs:mins), mean (SD) and median (iqi) | xxx (xx) | xxx (xx) |
| **Male,** n (%) | xxx (xx) | xxx (xx) |
| **Age** (years), mean (SD) | xxx (xx) | xxx (xx) |
| median (iqi) | xxx (xx) | xxx (xx) |
| ≥80**,** n (%) | xxx (xx) | xxx (xx) |
| **Ethnicity** |  |  |
| Asian, n (%) | xxx (xx) | xxx (xx) |
| Other, n (%) | xxx (xx) | xxx (xx) |
| **Clinical features** |  |  |
| Systolic BP (mmHg), mean (SD) | xxx (xx) | xxx (xx) |
| Diastolic BP (mmHg), mean (SD) | xxx (xx) | xxx (xx) |
| Heart rate (beats per minute), mean (SD) | xxx (xx) | xxx (xx) |
| NIHSS score |  |  |
| Median (iqi) | xxx (xx) | xxx (xx) |
| ≥14 (n, %) | xxx (xx) | xxx (xx) |
| GCS score |  |  |
| Median (iqi) | xxx (xx) | xxx (xx) |
| Severe (3-8), n (%) | xxx (xx) | xxx (xx) |
| **Medical history** |  |  |
| Hypertension, n (%) | xxx (xx) | xxx (xx) |
| Currently treated hypertension, n (%) | xxx (xx) | xxx (xx) |
| Previous stroke (ischaemic, hemorrhagic or uncertain), n (%) | xxx (xx) | xxx (xx) |
| Coronary artery disease, n (%) | xxx (xx) | xxx (xx) |
| Other heart disease (valvular or other), n (%) | xxx (xx) | xxx (xx) |
| Evidence of atrial fibrillation, n (%) |  |  |
| Definite history of atrial fibrillation n (%) | xxx (xx) | xxx (xx) |
| Atrial fibrillation confirmed on ECG, n (%) | xxx (xx) | xxx (xx) |
| Diabetes mellitus, n (%) | xxx (xx) | xxx (xx) |
| Hypercholesterolemia, n (%) | xxx (xx) | xxx (xx) |
| Current smoker, n (%) | xxx (xx) | xxx (xx) |
| Pre-stroke function (mRS), n (%) |  |  |
| 0 no symptoms | xxx (xx) | xxx (xx) |
| 1 no significant disability | xxx (xx) | xxx (xx) |
| **Medications at time of admission** |  |  |
| Antihypertensive agent(s), n (%) | xxx (xx) | xxx (xx) |
| Warfarin anticoagulation, n (%) | xxx (xx) | xxx (xx) |
| Aspirin or other antiplatelet agent, n (%) | xxx (xx) | xxx (xx) |
| Statin or other lipid lowering agent, n (%) | xxx (xx) | xxx (xx) |
| **Brain imaging features*** |  |  |
| CT scan used, n (%) | xxx (xx) | xxx (xx) |
| MRI scan used, n (%) | xxx (xx) | xxx (xx) |
| Visible early ischemic changes, n (%) | xxx (xx) | xxx (xx) |
| Visible cerebral infarction, n (%) | xxx (xx) | xxx (xx) |
| Visible cerebral infarction with mass effect, n (%) | xxx (xx) | xxx (xx) |
| CT or MR angiogram show proximal occlusion, n (%) | xxx (xx) | xxx (xx) |
| **Final diagnosis at time of hospital separation** |  |  |
| Non-stroke, n (%) | xxx (xx) | xxx (xx) |
| Presumed stroke pathology, n (%) |  |  |
| Large artery occlusion due to significant atheroma | xxx (xx) | xxx (xx) |
| Small vessel or perforating vessel lacunar disease | xxx (xx) | xxx (xx) |
| Cardio-embolic | xxx (xx) | xxx (xx) |
| Other or uncertain aetiology | xxx (xx) | xxx (xx) |

*Data based on clinician reported findings. Detailed analysis of brain imaging through central expert review to be undertaken in separate papers

**Table 2: Use of alteplase and management details from randomisation to Day 7**

|  | Low dose  (n=xxx) |  | Standard dose  (n=xxx) |  | P value |
| --- | --- | --- | --- | --- | --- |
| **Thrombolysis treatment** |  |  |  |  |  |
| Body weight |  |  |  |  |  |
| Patients with estimated body weight prior to alteplase, n (%) | xxx | xx | xxx | xx | 0.xxx |
| Estimated measurement prior to alteplase use, mean (SD) | xxx | xx | xxx | xx | 0.xxx |
| Direct measured body weight prior to alteplase use, mean (SD) | xxx | xx | xxx | xx | 0.xxx |
| Direct measurement body weight after alteplase use, mean (SD) |  |  |  |  |  |
| Alteplase administration |  |  |  |  |  |
| Any given, n (%) | xxx | xx | xxx | xx |  |
| Bolus dose (mg), mean (SD) | xxx | xx | xxx | xx | 0.xxx |
| Infusion over 60 mins dose (mg), mean (SD) | xxx | xx | xxx | xx | 0.xxx |
| Patients outside dose range, n (%) |  |  |  |  |  |
| Time from randomisation to treatment (mins), median (iqr) | xxx | xx | xxx | xx | 0.xxx |
| Time from stroke onset to treatment (mins), median (iqr) | xxx | xx | xxx | xx | 0.xxx |
| **Management** |  |  |  |  |  |
| Endovascular clot retrieval used, n (%) | xxx | xx | xxx | xx | 0.xxx |
| Any intravenous BP lowering treatment in first 24 hours, n (%) | xxx | xx | xxx | xx | 0.xxx |
| Any intravenous BP lowering treatment in days 2-7, n (%) | xxx | xx | xxx | xx | 0.xxx |
| Systolic BP at 24 hours, mmHg, mean (SD) | xxx | xx | xxx | xx | 0.xxx |
| Intubation and ventilation, n (%) | xxx | xx | xxx | xx | 0.xxx |
| Fever occurrence, n (%) | xxx | xx | xxx | xx | 0.xxx |
| Fever treated, n (%) | xxx | xx | xxx | xx | 0.xxx |
| Nasogastric feeding given, n (%) | xxx | xx | xxx | xx | 0.xxx |
| Patient mobilized by therapist, n (%) | xxx | xx | xxx | xx | 0.xxx |
| Compression stockings used, n (%) | xxx | xx | xxx | xx | 0.xxx |
| Subcutaneous heparin used, n (%) | xxx | xx | xxx | xx | 0.xxx |
| Any antithrombotic agent (antiplatelet or heparin) used in first 24 hours, n (%) | xxx | xx | xxx | xx | 0.xxx |
| Intravenous traditional Chinese medicine administered, n (%) | xxx | xx | xxx | xx | 0.xxx |
| Intravenous steroids administered, n (%) | xxx | xx | xxx | xx | 0.xxx |
| Hemicraniectomy performed, n (%) | xxx | xx | xxx | xx | 0.xxx |
| Any neurosurgery performed, n (%) | xxx | xx | xxx | xx | 0.xxx |
| Any stroke unit admission, n (%) | xxx | xx | xxx | xx | 0.xxx |
| Any intensive care unit admission, n (%) | xxx | xx | xxx | xx | 0.xxx |
| Any rehabilitation given, n (%) | xxx | xx | xxx | xx | 0.xxx |
| Decision to withdrawal active care, n (%) | xxx | xx | xxx | xx | 0.xxx |

*Definitions for protocol violation over alteplase dose: low-dose outside 0.6-0.75 mg/kg range; standard-dose outside of 0.75mg/kg range

**Table 3: Primary and key secondary outcomes at 90 days, by intention to treat, except where specified**

|  | Low dose (n=xxx) | Standard dose (n=xxx) | Odds ratio (95% CI) | P-value^*^ |
| --- | --- | --- | --- | --- |
|  | n (%) | n (%) |  |  |
| **Death or major disability (mRS score 2+3+4+5+6)** |  |  |  |  |
| Unadjusted | xxx (xx) | xxx (xx) | xxx (xxx-xxx) | 0.xxx |
| Adjusted^2^ | xxx (xx) | xxx (xx) | xxx (xxx-xxx) | 0.xxx |
| Per protocol | xxx (xx) | xxx (xx) | xxx (xxx-xxx) | 0.xxx |
| **Dead or disability** (mRS score 3+4+5+6) | xxx (xx) | xxx (xx) | xxx (xxx-xxx) | 0.xxx |
| **mRS categories** |  |  | xxx (xxx-xxx)† | 0.xxx |
| 0 | xxx (xx) | xxx (xx) |  |  |
| 1 | xxx (xx) | xxx (xx) |  |  |
| 2 | xxx (xx) | xxx (xx) |  |  |
| 3 | xxx (xx) | xxx (xx) |  |  |
| 4 | xxx (xx) | xxx (xx) |  |  |
| 5 | xxx (xx) | xxx (xx) |  |  |
| 6 (death before 90 days) | xxx (xx) | xxx (xx) | xxx (xxx-xxx) | 0.xxx |
| **Symptomatic intracerebral hemorrhage** |  |  |  |  |
| SITS-MOST criteria | xxx (xx) | xxx (xx) | xxx (xxx-xxx) | 0.xxx |
| NINDS criteria | xxx (xx) | xxx (xx) | xxx (xxx-xxx) | 0.xxx |
| ECASS2 criteria | xxx (xx) | xxx (xx) | xxx (xxx-xxx) | 0.xxx |
| Clinician-reported | xxx (xx) | xxx (xx) | xxx (xxx-xxx) | 0.xxx |
| Fatal (<7 days) | xxx (xx) | xxx (xx) | xxx (xxx-xxx) | 0.xxx |
| **Any intracerebral hemorrhage** | xxx (xx) | xxx (xx) | xxx (xxx-xxx) | 0.xxx |
| **Death or neurological deterioration in first 7 days** | xxx (xx) | xxx (xx) | xxx (xxx-xxx) | 0.xxx |

^*^Chi-square test or, if an expected cell count is lower than 5, Fisher’s exact test. If the total number of events is 0 the test is not required Chi-square test

†ordinal logistic regression analysis, adjusting for site, time from stroke onset to randomization, NIHSS as a continuous variable, age, sex, ethnicity, pre-morbid mRS (0 or 1), pre-morbid use of aspirin, other antiplatelet agent or warfarin, and any history of stroke, coronary artery disease, diabetes mellitus, and atrial fibrillation

**Table 4: Other secondary outcomes at 90 days**

|  | Low dose (n=xxx) |  | Standard dose (n=xxx) |  | Odds ratio  (95% CI)* | P-value† |
| --- | --- | --- | --- | --- | --- | --- |
|  | n | % | n | % |  |  |
| **Primary cause of death** |  |  |  |  |  |  |
| Direct effects of primary event | xxx | xx | xxx | xx | xxx (xx –xx) | 0.xxx |
| Acute intracerebral hemorrhage | xxx | xx | xxx | xx | xxx (xx –xx) | 0.xxx |
| Recurrent stroke | xxx | xx | xxx | xx | xxx (xx –xx) | 0.xxx |
| Intracerebral hemorrhage | xxx | xx | xxx | xx |  |  |
| Ischemic stroke | xxx | xx | xxx | xx |  |  |
| Undifferentiated stroke | xxx | xx | xxx | xx |  |  |
| Acute MI/coronary event | xxx | xx | xxx | xx | xxx (xx –xx) | 0.xxx |
| Other vascular | xxx | xx | xxx | xx | xxx (xx –xx) | 0.xxx |
| Non-vascular | xxx | xx | xxx | xx | xxx (xx –xx) | 0.xxx |
| **EQ5D score** |  |  |  |  |  |  |
| Problems with mobility – no./total no. (%) | xxx | xx | xxx | xx | xxx (xx –xx) | 0.xxx |
| Problems with self-care – no./total no. (%) | xxx | xx | xxx | xx | xxx (xx –xx) | 0.xxx |
| Problems with usual activities - no./total no. (%) | xxx | xx | xxx | xx | xxx (xx –xx) | 0.xxx |
| Problems with pain/discomfort - no./total no. (%) | xxx | xx | xxx | xx | xxx (xx –xx) | 0.xxx |
| Problems with anxiety/depression - no./total no. (%) | xxx | xx | xxx | xx | xxx (xx –xx) | 0.xxx |
| Overall health utility - mean±SD | xxx | xx | xxx | xx | xxx (xx –xx) | 0.xxx |
| **Living at home** | xxx | xx | xxx | xx | xxx (xx –xx) | 0.xxx |
| **Duration of initial hospitalization** | xxx | xx | xxx | xx | xxx (xx –xx) | 0.xxx |

*Chi-square test or, if an expected cell count is lower than 5, Fisher’s exact test. If total number of events is 0, the test is not required

†Chi-square test or, if an expected cell count is lower than 5, Fisher’s exact test.

**Table 5: Serious adverse events (SAEs) during follow-up**

|  | Low dose (n=xxx) |  | Standard dose (n=xxx) |  | Odds ratio (95% CI)* | P-value† |
| --- | --- | --- | --- | --- | --- | --- |
|  | n | % | n | % |  |  |
| **All SAEs** |  |  |  |  |  |  |
| # of events (including deaths) | xxx | xx | xxx |  | NA | NA |
| # of subjects with any SAE | xxx | xx | xxx | xx |  |  |
| Fatal SAE | xxx | xx | xxx | xx |  |  |
| **By category** |  |  |  |  |  |  |
| Neurological deterioration in the first 24 hours | xxx | xx | xxx | xx |  |  |
| Neurological deterioration in the first 72 hours | xxx | xx | xxx | xx |  |  |
| Death from stroke in first 7 days | xxx | xx | xxx | xx |  |  |
| Death or neurological deterioration in first 7 days | xxx | xx | xxx | xx |  |  |
| Symptomatic intracerebral hemorrhage | xxx | xx | xxx | xx |  |  |
| Major extracranial hemorrhage | xxx | xx | xxx | xx |  |  |
| Ischemic stroke | xxx | xx | xxx | xx |  |  |
| Undifferentiated stroke | xxx | xx | xxx | xx |  |  |
| Acute coronary event | xxx | xx | xxx | xx |  |  |
| Other vascular | xxx | xx | xxx | xx |  |  |
| Non-vascular | xxx | xx | xxx | xx |  |  |
| Pneumonia | xxx | xx | xxx | xx |  |  |
| Sepsis | xxx | xx | xxx | xx |  |  |
| Fracture | xxx | xx | xxx | xx |  |  |
| Other non-vascular | xxx | xx | xxx | xx |  |  |
| Angioedema | xxx | xx | xxx | xx |  |  |
| Other SAE | xxx | xx | xxx | xx |  |  |

Counts correspond to the number of subjects who experienced a specific SAE with the exception of the first row. Denominators are all subjects randomized.

* if feasible and test performed

†Chi-square test or, if an expected cell count is lower than 5, Fisher’s exact test. If total number of events is 0 the test is not required.

NB: Similar tables per type of SAE (fatal vs nonfatal) may also be produced.

**Table 6: Subgroup analyses – to be presented as a forest plot**

|  | Low dose (n=xxx) | Standard dose (n=xxx) | Odds ratio (95% CI) | P-value* |
| --- | --- | --- | --- | --- |
|  | n (%) | n (%) |  |  |
| **Age** |  |  |  |  |
| <65 years | xxx (xx) | xxx (xx) | xxx (xxx-xxx) | 0.xxx |
| ≥65 years | xxx (xx) | xxx (xx) | xxx (xxx-xxx) |  |
| **Sex** |  |  |  |  |
| Male | xxx (xx) | xxx (xx) | xxx (xxx-xxx) | 0.xxx |
| Female | xxx (xx) | xxx (xx) | xxx (xxx-xxx) |  |
| **Ethnicity** |  |  |  |  |
| Asian | xxx (xx) | xxx (xx) | xxx (xxx-xxx) | 0.xxx |
| Non-Asian | xxx (xx) | xxx (xx) | xxx (xxx-xxx) |  |
| **Time to randomization** |  |  |  |  |
| <3 hours | xxx (xx) | xxx (xx) | xxx (xxx-xxx) | 0.xxx |
| ≥3 hours | xxx (xx) | xxx (xx) | xxx (xxx-xxx) |  |
| **Baseline systolic BP** |  |  |  |  |
| Below overall mean | xxx (xx) | xxx (xx) | xxx (xxx-xxx) | 0.xxx |
| Above overall mean | xxx (xx) | xxx (xx) | xxx (xxx-xxx) |  |
| **Baseline NIHSS score** |  |  |  |  |
| Below overall median | xxx (xx) | xxx (xx) | xxx (xxx-xxx) | 0.xxx |
| Above overall median | xxx (xx) | xxx (xx) | xxx (xxx-xxx) |  |
| **Final diagnosis of AIS** |  |  |  |  |
| Large artery atheroma occlusion | xxx (xx) | xxx (xx) | xxx (xxx-xxx) | 0.xxx |
| Small vessel disease | xxx (xx) | xxx (xx) | xxx (xxx-xxx) |  |
| Cardio-embolic | xxx (xx) | xxx (xx) | xxx (xxx-xxx) |  |
| Other definite or uncertain pathology | xxx (xx) | xxx (xx) | xxx (xxx-xxx) |  |
| **Cerebral infarction on CT scan** |  |  |  |  |
| Yes | xxx (xx) | xxx (xx) | xxx (xxx-xxx) | 0.xxx |
| No | xxx (xx) | xxx (xx) | xxx (xxx-xxx) |  |
| **Pre-morbid use of aspirin** |  |  |  |  |
| Yes | xxx (xx) | xxx (xx) | xxx (xxx-xxx) | 0.xxx |
| No | xxx (xx) | xxx (xx) | xxx (xxx-xxx) |  |
| **Evidence of atrial fibrillation** |  |  |  |  |
| Yes | xxx (xx) | xxx (xx) | xxx (xxx-xxx) | 0.xxx |
| No | xxx (xx) | xxx (xx) | xxx (xxx-xxx) |  |

**Figure 1: Flow diagram of ENCHANTED alteplase dose arm based on CONSORT 2010**

## Enrolment

**A Per-protocol population** (analysed (n= )

- Excluded from analysis (give reasons) (n= )

**B Intention to treat population** (analysed n= )

- Excluded from analysis (give reasons) (n= )

## Follow-up (7 day)

## Follow-up (28 day)

## Follow-Up (90 day)

Alive at 90 days, disability known (n= )

Alive at 90 days, disability unknown (n= )

Not known to be dead, disability unknown (n=)

Dead <90 days (n=)

Alive at 90 days, disability known (n= )

Alive at 90 days, disability unknown (n= )

Not known to be dead, disability unknown (n=)

Dead <90 days (n=)

Alive at 28 days, disability known (n= )

Alive at 28 days, disability unknown (n= )

Not known to be dead, disability unknown (n=)

Dead <28 days (n=)

Alive at 7 days, disability known (n= )

Alive at 7 days, disability unknown (n= )

Not known to be dead, disability unknown (n=)

Dead <7 days (n=)

Alive at 28 days, disability known (n= )

Alive at 28 days, disability unknown (n= )

Not known to be dead, disability unknown (n=)

Dead <28 days (n=)

Alive at 7 days, disability known (n= )

Alive at 7 days, disability unknown (n= )

Not known to be dead, disability unknown (n=)

Dead <7 days (n=)

## Analysis plan

**B Intention to treat population** (analysed n= )

- Excluded from analysis (give reasons) (n= )

**A Per-protocol population** (analysed n= )

- Excluded from analysis (give reasons) (n= )

## Allocation

Allocated to intervention (n= )

Did not receive intervention (n= )

Allocated to intervention (n= )

Did not receive intervention (n= )

**Randomized** (n= )

**Figure 2: Odds ratios (95% confidence intervals) for the primary endpoint (low-dose compared to standard-dose alteplase)**

Favours

standard-dose alteplase

Favours

low-dose alteplase

1.14

1.0

A: ITT population - unadjusted

B: ITT population – adjusted^*^

C: ITT population shift analysis of mRS - adjusted

D: PP population – adjusted^*^

2.0

0

Odds ratio

*adjusting for site, time from stroke onset to randomization, NIHSS as a continuous variable, age, sex, ethnicity, pre-morbid mRS (0 or 1), pre-morbid use of aspirin, other antiplatelet agent or warfarin, and any history of stroke, coronary artery disease, diabetes mellitus, and atrial fibrillation

**Figure 2: mRS outcome at 90 days by treatment group**

0

2

4

6

5

3

1

xx%

xx%

xx%

xx%

xx%

xx%

xx%

xx%

xx%

xx%

xx%

xx%

xx%

xx%

1

2

3

4

5

6

0

**Patients_(%)**

**xx refer to percentage values in each level of mRS**

Unadjusted common odds ratio for improvement of 1 point on the mRS is XX (95% CI X.XX to X.XX).

**Figure 3: Effect of time of alteplase on an excellent outcome (mRS 0-1)**

**Odds ratio**

**1.0**

**Time from stroke onset to treatment (hours)**

**Appendix 3**

**Additional tables**

**Table 1: Compliance with trial treatment protocol**

|  | Low dose (n=xxx) | Standard dose (n=xxx) |
| --- | --- | --- |
|  | N (%)* | N (%)* |
| **Randomisation violations** |  |  |
| Age <18 years | xxx (xx) | xxx (xx) |
| Acute stroke syndrome not ischaemic stroke | xxx (xx) | xxx (xx) |
| Dependent pre-stroke | xxx (xx) | xxx (xx) |
| Significant comorbid condition | xxx (xx) | xxx (xx) |
| Systolic blood pressure >185 mmHg | xxx (xx) | xxx (xx) |
| Other | xxx (xx) | xxx (xx) |
| **Treatment compliance** |  |  |
| Alteplase given beyond 4.5 hours | xxx (xx) | xxx (xx) |
| Alteplase treatment protocol not followed | xxx (xx) | xxx (xx) |
| **Outcome assessment** |  |  |
| In-person or telephone assessment of 90 day outcome | xxx (xx) | xxx (xx) |
| Assessor predicted treatment allocation | xxx (xx) | xxx (xx) |

*denominator will vary for violations and compliance/outcomes

**Table 2: Other categorisation of baseline characteristics,** n (%)

|  | Low-dose group  (n=xxx) | Standard-dose group  (n=xxx) |
| --- | --- | --- |
| **Age,** years |  |  |
| 18-40 | xxx (xx) | xxx (xx) |
| 40-50 | xxx (xx) | xxx (xx) |
| 50-60 | xxx (xx) | xxx (xx) |
| 60-70 | xxx (xx) | xxx (xx) |
| 70-80 | xxx (xx) | xxx (xx) |
| 80-90 | xxx (xx) | xxx (xx) |
| >90 | xxx (xx) | xxx (xx) |
| ≥80 | xxx (xx) | xxx (xx) |
| **Region of recruitment** |  |  |
| UK/Europe/Australia | xxx (xx) | xxx (xx) |
| China | xxx (xx) | xxx (xx) |
| Other Asian | xxx (xx) | xxx (xx) |
| South America | xxx (xx) | xxx (xx) |
| **NIHSS score** |  |  |
| 0-5 | xxx (xx) | xxx (xx) |
| 6-10 | xxx (xx) | xxx (xx) |
| 11-15 | xxx (xx) | xxx (xx) |
| 16-20 | xxx (xx) | xxx (xx) |
| 21-35 | xxx (xx) | xxx(xx) |
| **Time to randomisation, hr** |  |  |
| 0-1 | xxx (xx) | xxx (xx) |
| 1-2 | xxx (xx) | xxx (xx) |
| 2-3 | xxx (xx) | xxx (xx) |
| 3-4 | xxx (xx) | xxx (xx) |
| >4 | xxx (xx) | xxx(xx) |
| **Blood glucose at randomisation, mmol/L** |  |  |
| ≤5 | xxx (xx) | xxx (xx) |
| 6-7 | xxx (xx) | xxx (xx) |
| ≥8 | xxx (xx) | xxx(xx) |
| **Medical history** |  |  |
| Previous ischaemic stroke | xxx (xx) | xxx (xx) |
| Previous intracerebral haemorrhage | xxx (xx) | xxx (xx) |
| Previous stroke of unknown type | xxx (xx) | xxx (xx) |
| Documented extra-cranial vascular disease | xxx (xx) | xxx (xx) |
| Documented intra-cranial vascular disease | xxx (xx) | xxx (xx) |
| Co-morbid coagulopathy or liver disease | xxx (xx) | xxx (xx) |

**Table 3: PP population - baseline characteristics**

|  | Low-dose group  (n=xxx) | Standard-dose group  (n=xxx) |
| --- | --- | --- |
| **Time from stroke onset to randomisation** (hrs:mins), mean (SD) and median (iqi) | xxx (xx) | xxx (xx) |
| **Male,** n (%) | xxx (xx) | xxx (xx) |
| **Age** (years), mean (SD) | xxx (xx) | xxx (xx) |
| median (iqi) | xxx (xx) | xxx (xx) |
| **≥80,** n (%) | xxx (xx) | xxx (xx) |
| **Ethnicity** |  |  |
| Asian, n (%) | xxx (xx) | xxx (xx) |
| Other, n (%) | xxx (xx) | xxx (xx) |
| **Clinical features** |  |  |
| Systolic BP (mmHg), mean (SD) | xxx (xx) | xxx (xx) |
| Diastolic BP (mmHg), mean (SD) | xxx (xx) | xxx (xx) |
| Heart rate (beats per minute), mean (SD) | xxx (xx) | xxx (xx) |
| NIHSS score |  |  |
| Median (iqi) | xxx (xx) | xxx (xx) |
| ≥14 (n, %) | xxx (xx) | xxx (xx) |
| GCS score |  |  |
| Median (iqi) | xxx (xx) | xxx (xx) |
| Severe (3-8), n (%) | xxx (xx) | xxx (xx) |
| **Medical history** |  |  |
| Hypertension, n (%) | xxx (xx) | xxx (xx) |
| Currently treated hypertension, n (%) | xxx (xx) | xxx (xx) |
| Previous stroke (ischaemic, haemorrhagic or uncertain), n (%) | xxx (xx) | xxx (xx) |
| Coronary artery disease, n (%) | xxx (xx) | xxx (xx) |
| Other heart disease (valvular or other), n (%) | xxx (xx) | xxx (xx) |
| Evidence of atrial fibrillation, n (%) |  |  |
| Definite history of atrial fibrillation n (%) | xxx (xx) | xxx (xx) |
| Atrial fibrillation confirmed on ECG, n (%) | xxx (xx) | xxx (xx) |
| Diabetes mellitus, n (%) | xxx (xx) | xxx (xx) |
| Hypercholesterolaemia, n (%) | xxx (xx) | xxx (xx) |
| Current smoker, n (%) | xxx (xx) | xxx (xx) |
| Pre-stroke function (mRS), n (%) |  |  |
| 0 no symptoms | xxx (xx) | xxx (xx) |
| 1 no significant disability | xxx (xx) | xxx (xx) |
| **Medications at time of admission** |  |  |
| Antihypertensive agent(s), n (%) | xxx (xx) | xxx (xx) |
| Warfarin anticoagulation, n (%) | xxx (xx) | xxx (xx) |
| Aspirin or other antiplatelet agent, n (%) | xxx (xx) | xxx (xx) |
| Statin or other lipid lowering agent, n (%) | xxx (xx) | xxx (xx) |
| **Brain imaging features** |  |  |
| CT scan used, n (%) | xxx (xx) | xxx (xx) |
| MRI scan used, n (%) | xxx (xx) | xxx (xx) |
| Visible early ischaemic changes, n (%) | xxx (xx) | xxx (xx) |
| Visible cerebral infarction, n (%) | xxx (xx) | xxx (xx) |
| Visible cerebral infarction with mass effect, n (%) | xxx (xx) | xxx (xx) |
| CT or MR angiogram show proximal occlusion, n (%) | xxx (xx) | xxx (xx) |
| **Final diagnosis at time of hospital separation** |  |  |
| Non-stroke, n (%) |  |  |
| migraine | xxx (xx) | xxx (xx) |
| seizure | xxx (xx) | xxx (xx) |
| functional weakness | xxx (xx) | xxx (xx) |
| syncope | xxx (xx) | xxx (xx) |
| other | xxx (xx) | xxx (xx) |
| Presumed stroke pathology, n (%) |  |  |
| Large artery occlusion due to significant atheroma | xxx (xx) | xxx (xx) |
| Small vessel or perforating vessel lacunar disease | xxx (xx) | xxx (xx) |
| Cardio-emboli | xxx (xx) | xxx (xx) |
| Other or uncertain aetiology |  |  |
|  |  |  |

**Appendix 4**

**Proposed content and timing of primary and subsequent publications**

| **N** | **2016** |
| --- | --- |
| 1 | Main results paper: differential treatment effects of alteplase doses on primary and secondary efficacy and safety outcomes, and according to pre-specified subgroups |
| 2 | Further subgroup analysis: relation of treatment effects by time, ethnicity and age |
| 3 | Further subgroup analysis: differential treatment effects by proximal clot occlusion of CT or MRI angiography |
| 4 | Further subgroup analysis: differential treatment effects by proximal clot occlusion identified on transcranial Doppler, including time and completeness of recanalisation |
| 5 | Further subgroup analysis: differential treatment effects by baseline neurological severity |
| 6 | Differential treatment effects according to use of endovascular clot retrieval |
|  | **2017 and subsequent years** |
| 7 | Differential treatment effects of alteplase doses on degree of cerebral ischemic lesion identified on brain imaging |
| 8 | Predictors of intracerebral hemorrhage |
| 9 | Clinical and imaging predictors of poor outcome |
| 10 | Health economic analysis: estimating cost-effectiveness by alteplase dose |
| 11 | Determinants of HRQoL and influence of age, sex, ethnicity and level of disability |
| 12 | Determinants of presentation and treatment times in AIS |
| 13 | Accuracy of estimation of body weight and influence of age, sex, ethnicity and severity |
| 14 | Regional variation in the management of AIS |
| 15 | Frequency, predictors and prognostic significance of seizures after stroke |
| 16 | Effects of alteplase in stroke mimics |
| 17 | Relation of conventional mRS to simplified mRS, and according to in-person versus telephone, in assessing outcome from AIS |
| 18 | Patterns of recovery according to mRS scores over 7, 28 and 90 days follow-up |
| 19 | Structural imaging descriptive analyses and correlation of perfusion imaging with plain CT |
| 20 | Clinical-radiological correlations of baseline imaging and clinical and pathological classifications |
| 21 | Interaction of treatment effect on primary outcome with centre experience and duration of participation and number of patients contributed in the trial |
| 22 | Inclusion of data in systematic reviews/meta-analyses including the Blood pressure in Acute Stroke Collaboration (BASC), Cochrane Stroke Group database of thrombolytic dose, and of brain imaging determinants of AIS outcome (J Wardlaw, University of Edinburgh) |
